# A hybrid random forest model for stroke risk prediction

**DOI:** 10.3389/fneur.2026.1822304

**Published:** 2026-04-10

**Authors:** Qixuan Lu, Shuo Li, Hongyuan Xu, Hui Shen, Yufei Wei

**Affiliations:** 1 Department of Cerebral Vascular Disease, Neurological Disease Center, Beijing Anzhen Hospital, Capital Medical University, Beijing, China; 2 Department of Neurology, Beijing Tiantan Hospital, Capital Medical University, Beijing, China; 3 China National Clinical Research Center for Neurological Diseases, Beijing, China

**Keywords:** machine learning, prediction model, random forest, risk stratification, stroke

## Abstract

**Objectives:**

Our study aims to develop a stroke risk prediction model by multiple machine learning algorithms and optimize the model as a stroke risk prediction tool.

**Methods:**

This retrospective multicenter study derived the original dataset from a high-quality health database. The dataset was incomplete and class imbalanced. Firstly, we eliminated extreme outliers and noises and imputed missing values by appropriate algorithms. We further used Synthetic Minority Over-sampling Technique to generate a balanced dataset. Secondly, we fitted seven algorithms to develop a machine learning-based prediction tool for clinical practice.

**Results:**

Overall, 35,859 participants were included, of whom 781 (2.2%) experienced a stroke. The random forest model demonstrated the best performance with high predictive value and discrimination ability. For stroke risk prediction, the AUC of the best-performing model was 0.97.

**Conclusion:**

A new random forest algorithms-based stroke risk prediction model using easily obtainable data was developed and outperformed established models. Future studies should further validate and optimize the current model to assess its generalizability and promote the wide application. The utilization of proposed random forest algorithms as an individualized risk prediction model could facilitate the application of clinical practice guidelines and shared decision-making.

## 1 Introduction

Stroke remains a major health problem with high morbidity and mortality that affects millions of people worldwide. Over the past two decades, the annual incidence, prevalence, and mortality of stroke have shown a significant surge, contributing to the heavy burden of stroke globally. According to the Global Burden of Disease Study 2019, stroke remains the second-leading cause of death and the third-leading cause of death and disability combined in the world. In fact, over the last 20 years, there has been a notable increase in the absolute number of incident strokes by 70%, prevalent strokes by 85%, stroke-related fatalities by 43%, and disability-adjusted life years attributed to strokes by 32%. The latest data shows there were nearly 100 million prevalent strokes annually, and an estimated 12 million people suffer a stroke each year (1). The burden of stroke-related mortality and disability is especially heavy in low- and middle income countries (2, 3). Moreover, stroke mortality is rising even in high-income countries over the past years, with younger populations bearing increasing risk (4). Therefore, developing effective prevention strategies for stroke is essential for improving public health and relieving the heavy medical cost and family burden. As most risk factors of stroke are modifiable, raising attention to stroke prevention at a public health level is of pragmatic significance. To that end, developing effective prevention strategies for stroke constitutes an urgent need.

Stroke risk prediction models are useful in identifying high-risk individuals, which can raise awareness, empower the appropriate use of further diagnostic tests, and enable primary prevention with proper management of controllable risk factors, thereby reducing the incidence of stroke (5). By far, several risk assessment scoring systems have been developed. The Framingham Stroke Profile (FSP) is a tool for estimating 10-year stroke risk. The scores are used to assess an individual’s risk of stroke by considering a variety of risk factors, including age, diabetes, blood pressure, smoking status, and previous history of heart disease or stroke. The tool is using the Cox proportional hazards regression model to build (6). Although the initial FSP has been refined multiple times over the past years, the validity of the FSP to predict stroke risk among different ethnic groups is still not fully established (7, 8). And the target population of the profile was the subjects aged 55–84 years and without prior stroke which was fail to cover the general population (6). Recent validation studies have further demonstrated these limitations in contemporary populations (9, 10). Moreover, there are several prediction models developed with conventional statistical analysis methods using different stroke risk factors in recent years (11–13). However, most of these models focus on general cerebral vascular disease (CVD) risk rather than stroke-specific risk, and the performance of these prediction models have not been independently validated. QRISK2 is a widely used and validated tool for cardiovascular disease risk prediction which is recommended in clinical practice and updated annually (14).

Retaining most of the conventional risk factors, it added factors including ethnicity, rheumatoid arthritis, chronic renal disease, etc. QRISK2 is a well-established tool focused on predicting the risk of cardiovascular disease but fails to give an absolute risk of stroke separately. Besides that, although adding more comprehensive risk factors generally enhances the performance of the model, it also becomes more difficult to use in everyday clinical practice. QStroke is one alternative stroke prediction tool based on data from large population studies for the prediction of an individual’s risk of ischemic cerebrovascular diseases. QStroke improved prediction performance compared with other existing risk scoring methods, yet it only focuses on ischemic events (15). The Ischemic Cardiovascular Disease model is another widely used prediction tool, but it fails to cover all age groups (16).

In recent years, a few attempts were made to develop machine learning (ML)-based stroke/CVD risk prediction models (17–19). In comparison with traditional modeling methods (e.g., statistical logistic regression), ML enables the use of various algorithms to identify complex patterns and deeper relationships in data which can often achieve higher accuracy in predicting risks. ML can automatically select the most important features from a large pool of potential predictors and can be adapted to different types of data which may be challenging for traditional statistical methods. Previous studies demonstrated that ML is useful for modeling multifactorial events in the medical field, including predicting disease risk, medical image analysis, etc. Recent systematic reviews have confirmed the effectiveness of multiple ML algorithms, particularly ensemble methods such as random forest, for stroke prediction (20, 21). Hybrid ML algorithms (22) have much higher scalability, as they enable the incorporation of large numbers of features and parameters into the models (23). Traditional ML is prone to fall into the curse of dimensionality. If there are many features, the amount of data should be considerably large to properly train the model; otherwise, the chance of overfitting the model would be high. Hybrid ML has the potential to overcome these issues. Furthermore, there is increasingly concerned about handling imbalanced data sets in data mining. The typical imbalanced data issue occurs when there are significantly more samples in the majority class compared to the minority class. This can happen in both binary and multi-class classification problems. Such situations that there are significantly fewer patients with medical diagnoses compared to healthy individuals are common in the medical field, such as predicting the risk of tumors, A lot of effort has been made to handle the issue of imbalanced data. In terms of the algorithm, optimizing the algorithm structure design can enhance classification performance, while in terms of data, the primary approach involves constructing minority samples to increase the imbalance rate (24, 25). Thus, hybrid ML constitutes a promising method for outcome prediction and may be superior to classic logistic regression and traditional ML models.

Our study aims to design a model for predicting stroke based on risk variables in a large dataset using multiple machine learning algorithms. It’s hypothesized that hybrid ML could capture high-dimensional, non-linear correlations among multimodal clinical characteristics, and it could be used to create a risk stratification system that predicts stroke for individuals more accurately than the risk tools that are now available. The proposed model would be of potential to provide clinicians and researchers with an easy-to-use automatic tool for optimal stroke risk prediction and to facilitate the application of clinical practice guidelines and decision-making.

## 2 Methods

### 2.1 Data source, study population, and variables

We derived the original dataset from a high-quality health database from the United States Department of Health and Human Services, HealthData.gov in this retrospective multicenter study. The dataset contains 11 features, including subject identity (ID), gender, age, marital status, work type, residence type, hypertension, heart disease, average glucose level, body mass index (BMI), and smoking status. Data on stroke occurrence were obtained from the variables in the database. Subjects aged ≥18 years were recruited. This study was exempt from ethical review and informed consent was waived, as the data were publicly available and de-identified.

### 2.2 Processing of outliers and missing values

The dataset included some extreme outliers and noises that should be eliminated at the very beginning. Furthermore, the dataset was incomplete as around 30% of smoking status items and 3% of BMI items were missing. There are two commonly used missing value imputation methods: one is to impute with estimated special values, such as mean, median, and mode; another is to use algorithms. For continuous data (BMI), regression algorithms such as Support Vector Regression (SVR), Linear SVR, Random Forest Regression (RFR), Bayesian Ridge Regression (BRR), and Gradient Boosting Machine (GBM) were used. For discrete data (smoking status), classification algorithms such as K-Nearest Neighbor (kNN), Logistic Regression (LR), Decision Trees (DT), and Random Forest (RF) algorithms were used. To ensure the stability of the data, different strategies were compared by calculating the MSE or the accuracy, and the best one was chosen to impute the missing values. MSE is the average squared difference between the predicted and observed values and is used as the evaluation metric for regression models. MSE is defined as the Equation 1 below:

$$\mathrm{MSE}=\frac{1}{n}\sum_{i=1}^{n}{\left(Y_{i}-{\hat{Y}}_{i}\right)}^{2}$$ (1)

Where $Y_{i}$ denotes the actual number of positive samples in $i^{\mathrm{th}}$ data, ${\hat{Y}}_{i}$ represents the estimated result, and n is the number of samples in the dataset groups. The lower the value of MSE, the better the quality, and vice versa.

### 2.3 Processing of class imbalanced datasets

Among 35,859 subjects included in this study, 781 stroke events were recorded, only accounting for 2.2% of the total and much lower than 50%. Thus, the dataset was a typically imbalanced one. When the difference exists in the majority and minority samples is too large, the problem of overhigh accuracy and low classification ability of the classifier arises, and the trained classifier will not be able to fit the minority class well. To improve the classifier performance, re-sampling methods are commonly used to address the negative effect of imbalanced training datasets and to generate useful synthetic samples for the classification of imbalanced data. The current study used under-sampling and over-sampling techniques to handle the imbalanced dataset (26). The basic principle of under-sampling involves the random elimination of data points from the majority class until a state of balance is attained among the majority and minority classes. The over-sampling is to randomly choose the data points from the minority class and add them to the existing minority class (27).

SMOTE is a commonly used over-sampling technique. The principle of SMOTE is generating synthetic data by using the feature space similarities in the existing samples of the minority class (28), rather than simply duplicating the original minority samples, it is implemented in “feature space” rather than “data space”. Specifically, the SMOTE method synthesizes new samples by interpolating them near the selected minority samples.

For the generation of the new sample strategy, the SMOTE formula below was used (Equation 2).

$$x_{\mathrm{new}}=x+\mathrm{rand}(0,1)\times (\tilde{x}-x)$$ (2)

Where x is a randomly selected positive sample and $\tilde{x}$ is one of the nearest neighbors to x. Based on the formula and algorithmic rules, the SMOTE method is outlined in the following steps.

Initialize the N and k, where the N represents that *N*% of the samples will be oversampled and the k represents the k nearest forest neighbors of a particular sample.

Select a sample from the minority samples class whose data needs to be synthesized.

Randomly choose one of the k nearest neighbors from this sample.

According to Equation 2, calculate the difference between the considered sample and the randomly selected neighbors, multiply this difference by a random number between 0 and 1 to obtain the generated new sample, and add it to the minority samples class.

If *N*% of the samples in the minority samples class is manipulated, the process will stop. Otherwise, skips to step 2.

Unlike under-sampling, over-sampling does not result in information loss. We evaluated two re-sampling methods and selected the most appropriate sampling strategy for re-sampling data according to the accuracy, sensitivity, specificity, and G-mean of the model derived with the same algorithm. Eventually, a complete and balanced dataset was generated.

### 2.4 Machine learning algorithms

We fitted seven algorithms to construct the stroke risk prediction models, including LR, Gaussian Naive Bayes (GNB), Support Vector Classifier (SVC), Linear Support Vector Classifier (Linear SVC), DT, RF, and Multilayer Perceptron (MLP). The receiver operating characteristic (ROC) curve analysis was conducted, and the area under the curve (AUC) was used as the index of the prediction accuracy to compare the ROC curves. The best algorithm was selected by comparing accuracy, sensitivity, specificity, G-Mean, and AUC. Afterwards, the critical parameters were further calibrated to optimize the model.

### 2.5 Random forest

RF is widely used in processing classification and regression tasks (29). RF can handle data with multiple features and filter different features according to their importance. As a result, it has the potential to handle large amounts of variables and produce an accurate classifier. Besides, the RF model can maintain excellent performance in processing high-dimensional data because it injects a certain amount of randomness into each decision tree, which is demonstrated by using random features and random input selection. This is the key to the consistently lower generalization error of RF compared to other forests. Specifically, to improve classification accuracy, the randomness injected into the decision tree needs to minimize correlation while keeping strength. So each tree independently introduces random feature selection, while the samples used to build each tree in the forest are replaced randomly from the training samples, both of these make the RF relatively robust to outliers and noise (29).

In a decision tree, the dataset is divided into smaller subsets, and decision nodes and leaf nodes are generated simultaneously (30). Each tree acts as a classifier $\left\{y(x,\Theta_{n})|n=1,2,\ldots \right\}$ , where $\left\{\Theta_{n}\right\}$ is an independent and identically distributed random vector and each decision tree calculates the highest probability class based on the input x . RF is trained to combine multiple randomized decision trees and aggregate their predictions by averaging (31), thereby building a model with a better classification effect.

The process of computing and generating a vote for each tree based on the input data is as follows: Firstly, the decision trees are constructed by the Bootstrap method, which means that repeatedly and uniformly select K samples from the training data and use these samples to train K decision trees. Each K samples are chosen from the whole dataset, therefore there is overlap but not identical between the sample sets, so there is variability between the trained decision trees. Each tree is given the same weight, and the results are voted on separately. If the input data has P features, then each tree will be randomly assigned p≪P features. During the growth of the forest, no trees are pruned and p remains unchanged. This offers the advantage of significantly reducing the correlation between each decision tree, thereby improving its prediction accuracy on average. After the forest is constructed and trained as described above, each decision tree is individually calculated and voted to classify the new data. Finally, the RF model records all the votes, and the voting result category with the largest proportion is considered as the final output.

To improve the classification performance of the model, the Rao-1 algorithm was used to optimize the parameters of RF as Algorithm 1. The key to the Rao-1 algorithm is based on the stochastic interaction between the best and worst solutions in the aggregate and the candidate solutions, iteratively updating until the global optimum is found. The Rao-1 algorithm is an efficient and accurate optimization method that can complete the optimization process by setting only two basic control parameters, which are the population size and the number of iterations, and not requiring any other algorithm-specific parameters.

ALGORITHM 1 The optimization method.
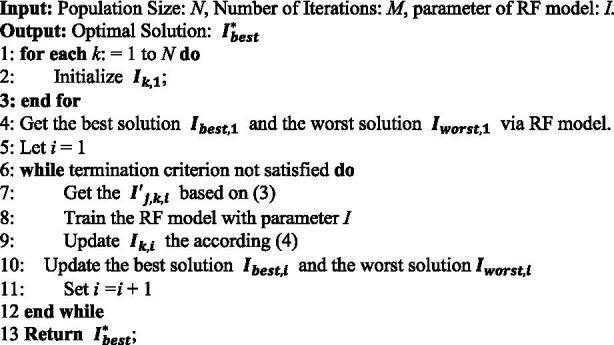

**Figure 1:**
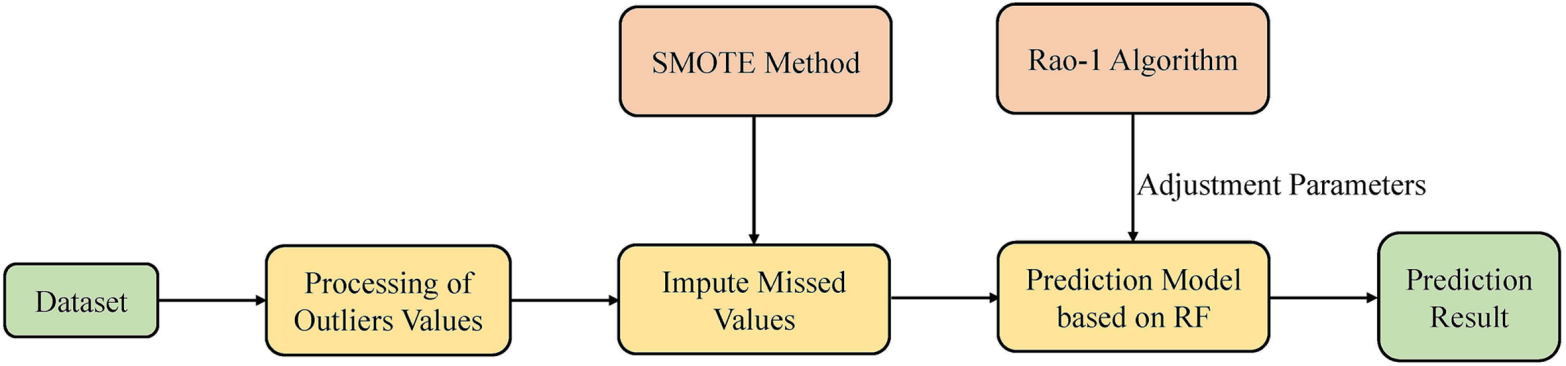
The overall framework.

The overall framework is shown in Figure 1.

The solution updating procedure of the Rao-1 algorithm is illustrated as Equations 3, 4:

$$I_{j,k,i}^{\prime }=I_{j,k,i}+r_{1,j,i}(I_{j,\text{best},i}-I_{j,\text{worst},i})$$ (3)

$$I\prime_{k,i+1}=\{\begin{array}{c} I\prime_{k,i}\;\;\text{if}\;F(I\prime_{k,i})\leq F(I_{k,i})\\ I_{k,i}\;\;\text{if}\;F(I\prime_{k,i})>F(I_{k,i}) \end{array}$$ (4)

Where $I_{j,\text{best},i}\;$ and $I_{j,\text{worst},i}$ are the values of the variable j for the best candidate and the worst candidate during the $i^{\mathrm{th}}$ iteration. $I\prime_{j,k,i}$ is the updated value of $I_{j,k,i}$ , and $r_{1,j,i}$ and $r_{2,j,i}\;$ are two random numbers of the $j^{\mathrm{th}}$ variable during the $i^{\mathrm{th}}$ iteration, within a value range of {0, 1}. The detailed steps of the Rao-1 method are described as follows: First, the public control parameters are initialized, which are the sample size, the number of variables and iterations. Then, the solutions with the best and worst results in all iterations are recorded. Next, the current solution is updated according to Equation 3 based on the recorded best solution, worst solution and candidate solution. Then calculate the objective function value for each updated solution and select the new optimal solution according to Equation 4. Finally, skip back to step 2 and continue repeating until the maximum number of iterations is attained.

## 3 Results

### 3.1 Data processing

After excluding the samples younger than 18 years old, 35,859 participants (mean age 49.3 ± 17.8 years, 38.8% males) were enrolled from the database. Among them, 781 (2.2%) subjects experienced a stroke. The reasonable reference value of the BMI is 10–50 kg/m^2^. Hence, those whose BMI was greater than 50 kg/m^2^ or less than 10 kg/m^2^ in the sample were excluded. In addition, the subject ID only serves as the subject’s identification and is a typical redundant term that needs to be excluded as an outlier. Table 1 shows the baseline characteristics of the participants according to the stroke status. Compared with subjects without stroke, subjects with stroke were older, were more likely to be male, had more vascular risk factors (history of hypertension and heart disease), and had a higher glucose level.

**Table 1:** Baseline characteristics of participants.

Characteristic	Total (*n* = 35,859)	With stroke (*n* = 781)	Without stroke (*n* = 35,078)	*p* value
Age, mean ± SD (years)	49.3 ± 17.8	68.3 ± 11.9	48.9 ± 17.6	<0.001
Male, *n* (%)	13,908 (38.8)	352 (45.1)	13,556 (38.7)	<0.001
History of hypertension, *n* (%)	4,056 (11.3)	200 (25.6)	3,856 (11.0)	<0.001
History of heart disease, *n* (%)	2058 (5.7)	177 (22.7)	1881 (5.4)	<0.001
Current smoker, *n* (%)	6,489 (22.6)	133 (20.8)	6,356 (22.7)	0.278
Married, *n* (%)	13,792 (48.1)	354 (55.5)	13,438 (47.9)	<0.001
Work type, *n* (%)				<0.001
Unemployed	58 (0.2)	0	58 (0.2)	
Private job	23,728 (66.2)	441 (56.5)	23,287 (66.4)	
Self-employed	6,694 (18.7)	251 (32.1)	6,443 (18.4)	
Government job	5,379 (15.0)	89 (11.4)	5,290 (15.1)	
Rural residence, *n* (%)	17,856 (49.8)	383 (49.0)	17,473 (49.8)	0.67
Glucose level, mean ± SD (mg/dL)	107.0 ± 45.7	129.8 ± 59.7	106.5 ± 45.2	<0.001
Body mass index, mean ± SD (kg/m^2^)	29.8 ± 6.4	29.7 ± 6.1	29.8 ± 6.4	0.66

Regarding the imputation of missing data, different strategies were utilized based on the characteristics of the variables. Algorithms were better than the special values to impute missing values of BMI and smoking status. Tables 2, 3 present the results of the comparison of different imputation strategies for BMI and smoking status. The model derived by GBM with the least MSE was chosen for the regression to fill the missing BMI values, and the model derived by LR with the highest accuracy was used for smoking status imputation.

**Table 2:** Comparison of different imputation strategies for BMI.

Metric	Special values			Algorithms				
	Mean	Median	Mode	SVR	Linear SVR	RFR	BRR	GBM
MSE	0.999	1.020	1.423	0.949	0.981	1.080	0.955	0.924

BMI, body mass index; BRR, Bayesian Ridge Regression; GBM, Gradient Boosting Machine; RFR, Random Forest Regression; SVR, Support Vector Regression.

**Table 3:** Comparison of different imputation strategies for smoking status.

Metric	Special values		Algorithms			
	Median	Mode	kNN	LR	RF	DT
Accuracy	0.513	0.513	0.466	0.521	0.424	0.400

DT, Decision Trees; kNN, K-Nearest Neighbor; LR, Logistic Regression; RF, Random Forest.

The results of comparisons among different re-sampling strategies are shown in Table 4. The under-sampling strategy resulted in the loss of a large number of negative samples, and the LR classifier using SMOTE sampling outperformed the under-sampling strategy (G-mean 0.782 vs. 0.771). In addition, the false positive rate and false negative rate were lower with the SMOTE sampling strategy compared with under-sampling. Therefore, the oversampling strategy of SMOTE was used for the generation of balanced samples.

**Table 4:** Comparison of different re-sampling strategies.

Re-sampling strategies	False positive	False negative	Accuracy	Sensitivity	Specificity	G-Mean
Original data	No positive	0.019	0.981	0.000	1.000	0.000
Under-sampling	0.242	0.209	0.773	0.815	0.729	0.771
SMOTE	0.232	0.196	0.784	0.743	0.823	0.782

### 3.2 Comparison of different machine learning algorithms

The details on the performance of models derived using each ML algorithm are shown in Table 5. The false positive, false negative, accuracy, sensitivity, specificity, and G-Mean of each model were compared.

**Table 5:** The false positive, false negative, accuracy, sensitivity, specificity, and G-Mean of each model.

Algorithm	False positive	False negative	Accuracy	Sensitivity	Specificity	G-Mean
LR	0.206	0.194	0.800	0.814	0.786	0.800
MLP	0.217	0.206	0.788	0.800	0.776	0.788
GNB	0.231	0.206	0.781	0.806	0.756	0.780
SVC	0.262	0.194	0.769	0.823	0.716	0.768
Linear SVC	0.208	0.215	0.789	0.786	0.791	0.789
DT	0.070	0.036	0.946	0.966	0.926	0.946
RF	0.045	0.020	0.967	0.981	0.953	0.967

DT, Decision Trees; GNB, Gaussian Naive Bayes; LR, Logistic Regression; MLP, Multilayer Perceptron; RF, Random Forest; SVC, Support Vector Classifier.

Out of the seven ML algorithms, RF and DT algorithms demonstrated notably superior prediction effectiveness when compared to other algorithms. Specifically, RF had the highest G-Mean and the lowest rates of false positives and false negatives, ultimately resulting in superior classification results.

**Figure 2:**
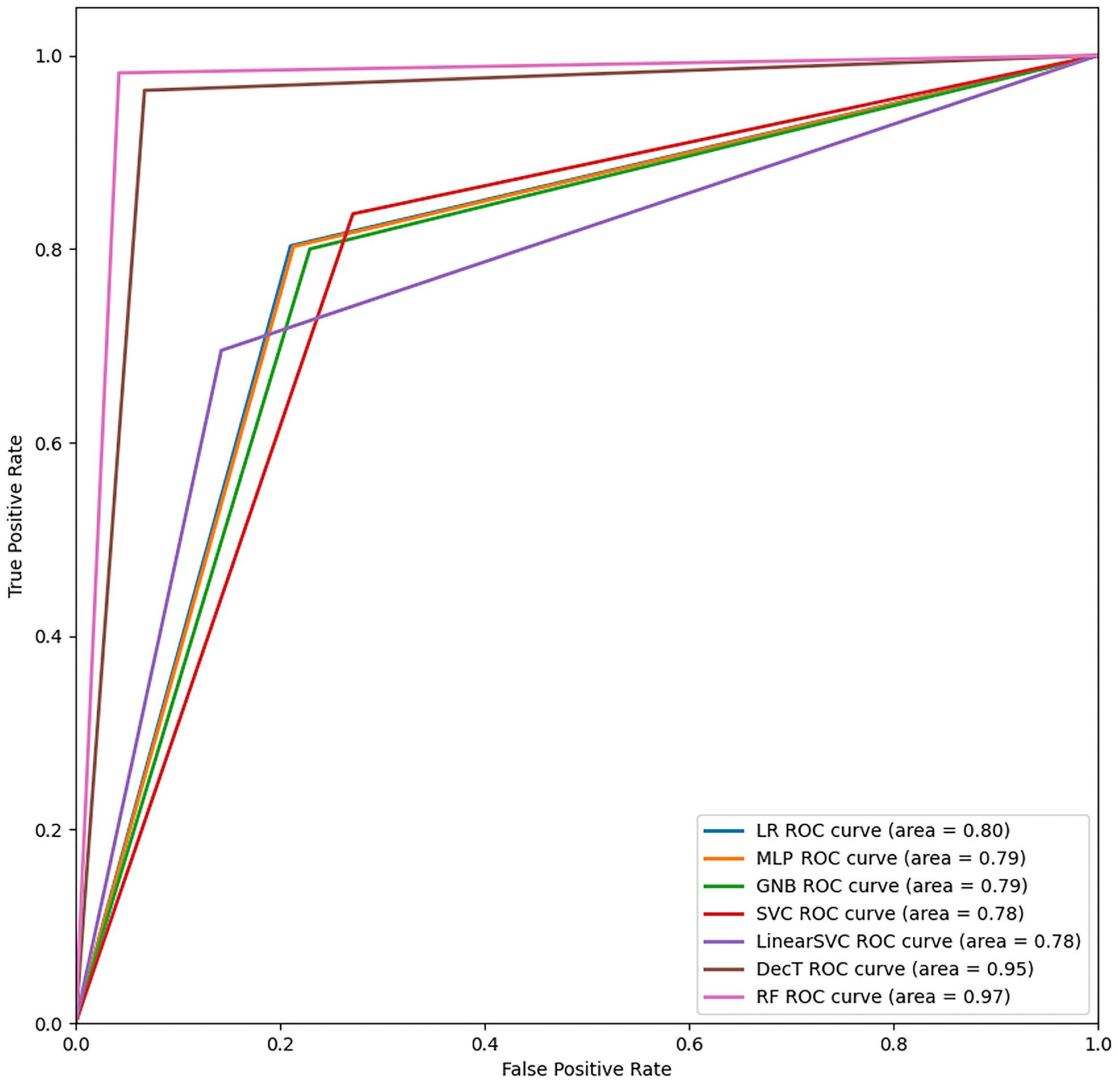
The ROC curves of each machine learning model.

Figure 2 illustrates the ROC curves of the stroke risk prediction models, providing information on the sensitivity, specificity, accuracy, and AUC of each model. The AUC of the models was as follows: 0.80 (LR), 0.79 (MLP), 0.79 (GNB), 0.78 (SVC), 0.78 (Linear SVC), 0.95 (DT), and 0.97 (RF). Among the seven machine learning algorithms, RF had the greatest AUC, indicating better performance in the prediction of stroke risk compared with the other 6 algorithms.

### 3.3 Algorithm optimization

The comparison of the above models was based on the non-parameter adjustment of all algorithms. We further adjusted the parameters of the RF algorithm to enhance the model’s classification performance. {min_samples_leaf = 1, max_features = 3, n_estimators = 100} were chosen as the tuning parameters. The performance of the model was further improved after the parameter tuning (Table 6).

**Table 6:** The performance of RF model before and after the optimization.

Optimization	False positive	False negative	Accuracy	Sensitivity	Specificity	G-Mean
Before	0.045	0.020	0.967	0.981	0.953	0.967
After	0.042	0.013	0.972	0.988	0.956	0.972

RF, Random Forest.

## 4 Discussion

In the current study, we developed a hybrid ML approach to predict stroke risk using an incomplete and imbalanced physiological dataset. With the dynamic optimization of the hyperparameters of the ML model, we greatly reduced the false negative rate of the stroke prediction. The utilization of proposed RF algorithms as an individualized risk prediction model could facilitate the application of clinical practice guidelines and shared decision-making based on optimal stroke risk prediction.

There are many risk factors related to elevated stroke risk, and these risk factors can be categorized as non-modifiable (e.g., age, gender, race, and ethnicity) and modifiable (hypertension, heart disease, diabetes mellitus, obesity, tobacco use) (32). The prediction model in our study used parameters that are easily obtainable and provides a simple but useful tool for predicting the risk of stroke among the whole population. Early identification of individuals with increased stroke risk for further assessment in the clinic is determinant for stroke prevention and avoiding the subsequent progression into disability.

Several statistically derived risk prediction models have been widely used, such as the new FSP, QStroke, and Ischemic Cardiovascular Disease model (8, 15, 16). However, the performance of these prediction models remains controversial (33). The use of poorly calibrated models may underestimate the risk of stroke, leading to missed opportunities to provide professional medical advice for those who could benefit. Thus, the ongoing refinement and enhancement of prediction models are crucial to ensure accurate risk stratification and effective preventive care in the medical field. Additionally, most of these models focus on general CVD risk or only ischemic events rather than stroke-specific risk, and some are applicable to only a specific population and fail to cover all age groups. Early detection of stroke risk is a crucial step for efficient preventative treatment, and ML can be of great value for achieving this purpose. Several models based on ML techniques have been developed and proven to be more accurate and effective in predicting performance for risk of stroke or overall CVD compared with traditional Cox models. These machine learning models use a variety of algorithms and can analyze complex datasets with multiple variables, providing more accurate and personalized risk assessments (18, 34, 35). Chun et al. have developed a stroke risk prediction model in a large sample of Chinese adults without prior history of stroke (19). The results showed GBT provided the best discrimination for stroke risk prediction with an AUROC of 0.833 and 0.836 for the males and females respectively, which was outperformed by our model. However, such improvements were marginal, and future studies should assess their reproducibility through external validation.

It should be pointed out that the dataset was a typically imbalanced one with classification issues that could negatively impact the performance of ML algorithms (36, 37). Lately, there has been an increasing concern over the classification of imbalanced datasets. Due to the imbalanced distribution of the datasets, several potential solutions have been proposed, including data re-sampling and RF, in order to effectively handle the situation (38, 39). There are two classic resampling algorithms, SMOTE and Edited nearest neighbor (ENN). The SMOTE algorithm utilizes linear interpolation to increase the sample size of the minority class (40), whereas ENN decreases the sample size of the majority class by eliminating noise samples (41). RF method generates a new dataset using bootstrap sampling from the original data, which can mitigate the impact of imbalanced samples and improve the recognition rate of underrepresented minority samples. However, each approach has its limitations. The SMOTE algorithm may lead to distribution marginalization and the number of nearest neighbors needs to be set manually for data expansion (42). One well-recognized shortcoming of ENN is the limited number of deleted noise samples (43). For the RF method, the increase in the amount of imbalanced data compromises its class effects. In the current study, re-sampling methods, including under-sampling and over-sampling methods, were used to generate a new balanced dataset. After evaluating the above methods, we adopted SMOTE algorithm for re-sampling data. A new balanced sample is generated by SMOTE by random linear interpolation in order to expand the minority class and improve classification accuracy. However, the SMOTE approach may generate noise samples and boundary samples, which is a limitation of our study.

According to the findings, among the seven ML algorithms, the RF model demonstrated superior performance with high predictive value and discrimination ability. RF is an ensemble classifier that generally integrates a large number of decision trees. Ensemble learning can achieve better performance compared with a single learner. RF is one of the most widely used algorithms in medical information analyses, such as medical image analysis, prediction model building, and genomic profiling (44–46). RF algorithm performs well when dealing with highly correlated complex data, especially in datasets with feature dimensions much larger than the sample size, which may account for its better performance in the current study.

Our study developed a dynamically optimized hyperparametric ML model to predict stroke risk and outperformed the previous model. All the variables in the prediction model have been proven to be closely related to the occurrence of stroke and can be easily obtained in daily clinical practice. The proposed RF algorithms could be used as an individualized risk prediction model to guide clinical practice and facilitate decision-making based on optimal stroke risk prediction. However, the authenticity of the data in our study may be diminished due to the handling of the missing data and imbalanced samples. Therefore, our model’s clinical applicability and generalizability need to be externally validated with other datasets. Future studies need to validate the model in external datasets to assess its performance compared with other stroke prediction tools.

## 5 Conclusion

The proposed RF algorithms-based model requires only easily obtainable data and thus could be used as a screening tool to predict stroke risk effectively. It could benefit decision-making and guide clinical practice for stroke prevention. Future studies should further validate and optimize the current model to assess its generalizability and promote its wide application.

## Data Availability

The data analyzed in this study is subject to the following licenses/restrictions: the data presented in this study are available on request from the corresponding author. Requests to access these datasets should be directed to YW, wyfwyf315@126.com.
